# Effect of training-sample size and classification difficulty on the accuracy of genomic predictors

**DOI:** 10.1186/bcr2468

**Published:** 2010-01-11

**Authors:** Vlad Popovici, Weijie Chen, Brandon G Gallas, Christos Hatzis, Weiwei Shi, Frank W Samuelson, Yuri Nikolsky, Marina Tsyganova, Alex Ishkin, Tatiana Nikolskaya, Kenneth R Hess, Vicente Valero, Daniel Booser, Mauro Delorenzi, Gabriel N Hortobagyi, Leming Shi, W Fraser Symmans, Lajos Pusztai

**Affiliations:** 1 Bioinformatics Core Facility, Swiss Institute of Bioinformatics, Génopode Building, Quartier Sorge, Lausanne CH-1015, Switzerland; 2 Center for Devices and Radiological Health, US Food and Drug Administration, 10903 New Hampshire Ave WO62-3124, Silver Springs, MD 20993-0002, USA; 3 Nuvera Biosciences, 400 West Cummings Park, Woburn, MA 01801, USA; 4 GeneGo, Inc., 500 Renaissance Drive, St. Joseph, MI 49085, USA; 5 Department of Systems Biology, Vavilov Institute for General Genetics, Russian Academy of Sciences, Gubkina str. 3 korp. 1, Moscow 119333, Russia; 6 Department of Biostatistics, P.O. Box 301439, Houston, TX 77230-1439, USA; 7 Department of Breast Medical Oncology, P.O. Box 301439, Houston, TX 77230-1439, USA; 8 Swiss NCCR Molecular Oncology, Swiss Institute for Experimental Cancer Research (ISREC), School of Life Sciences, Ecole Polytechnique Fédérale de Lausanne, Lausanne CH-1015, Switzerland; 9 National Center for Toxicological Research, US Food and Drug Administration, Jefferson, AR 72079, USA; 10 Department of Pathology of the University of Texas M. D. Anderson Cancer Center, P.O. Box 301439, Houston, TX 77230-1439, USA

## Abstract

**Introduction:**

As part of the MicroArray Quality Control (MAQC)-II project, this analysis examines how the choice of univariate feature-selection methods and classification algorithms may influence the performance of genomic predictors under varying degrees of prediction difficulty represented by three clinically relevant endpoints.

**Methods:**

We used gene-expression data from 230 breast cancers (grouped into training and independent validation sets), and we examined 40 predictors (five univariate feature-selection methods combined with eight different classifiers) for each of the three endpoints. Their classification performance was estimated on the training set by using two different resampling methods and compared with the accuracy observed in the independent validation set.

**Results:**

A ranking of the three classification problems was obtained, and the performance of 120 models was estimated and assessed on an independent validation set. The bootstrapping estimates were closer to the validation performance than were the cross-validation estimates. The required sample size for each endpoint was estimated, and both gene-level and pathway-level analyses were performed on the obtained models.

**Conclusions:**

We showed that genomic predictor accuracy is determined largely by an interplay between sample size and classification difficulty. Variations on univariate feature-selection methods and choice of classification algorithm have only a modest impact on predictor performance, and several statistically equally good predictors can be developed for any given classification problem.

## Introduction

Gene-expression profiling with microarrays represents a novel tissue analytic tool that has been applied successfully to cancer classification, and the first generation of genomic prognostic signatures for breast cancer is already on the market [1-3]. So far, most of the published literature has addressed relatively simple classification problems, including separation of cancer from normal tissue, distinguishing between different types of cancers, or sorting cancers into good or bad prognoses [4]. The transcriptional differences between these conditions or disease states are often large compared with transcriptional variability within the groups, and therefore, reasonably successful classification is possible. The methodologic limitations and performance characteristics of gene expression based classifiers have not been examined systematically when applied to increasingly challenging classification problems in real clinical data sets.

The MicroArray Quality Control (MAQC) (MAQC Consortium project-II: a comprehensive study of common practices for the development and validation of microarray-based predictive models) breast cancer data set (Table 1) offers a unique opportunity to study the performance of genomic classifiers when applied across a range of classification difficulties.

**Table 1:** Patient characteristics in the training and validation sets

	Training set (*n* = 130)	Validation set (*n* = 100)	*P *value
Median age	51 years (28-79 years)	50 years (26-73 years)	
Race			0.804
Caucasian	85 (65%)	68 (68%)	
African American	13 (10%)	12 (12%)	
Asian	9 (7%)	7 (7%)	
Hispanic	21 (16%)	13 (13%)	
Mixed	2 (2%)	0	
Cancer histology			0.047
Invasive ductal (IDC)	119 (92%)	85 (85%)	
Mixed ductal/lobular (IDC/ILC)	8 (6%)	8 (8%)	
Invasive lobular (ILC)	1 (0.7%)	7 (7%)	
Others	2 (1.3%)	0	
Tumor size			0.643
T0	1 (1%)	2 (2%)	
T1	12 (9%)	8 (8%)	
T2	70 (54%)	62 (62%)	
T3	21 (16%)	13 (13%)	
T4	26 (20%)	15 (15%)	
Lymph node stage			0.935
N0	39 (30%)	27 (27%)	
N1	60 (46%)	47 (47%)	
N2	14 (11%)	13 (13%)	
N3	17 (13%)	13 (13%)	
Nuclear grade (BMN)			0.005
1	2 (2%)	11 (11%)	
2	52 (40%)	42 (42%)	
3	76 (58%)	47 (47%)	
Estrogen receptor			0.813
Estrogen receptor positive	80 (62%)	60 (60%)	
Estrogen receptor negative	50 (38%)	40 (40%)	
HER-2			< 0.001
HER-2 positive	33 (25%)	7 (7%)	
HER-2 negative	96 (74%)	93 (93%)	
Neoadjuvant therapy			0.005
Weekly T × 12 + FAC × 4	112 (86%)	98 (98%)	
3-Weekly T × 4 + FAC × 4	18 (14%)	2 (2%)	
Pathologic complete response (pCR)	33 (25%)	15 (15%)	0.055

Estrogen receptor: cases in which more than 10% of tumor cells stained positive for ER with immunohistochemistry (IHC) were considered positive. HER-2: cases that showed either 3+ IHC staining or had gene copy number greater than 2.0 were considered HER-2 "positive." T = paclitaxel; FAC = 5-fluorouracil, doxorubicin, and cyclophosphamide. The *P *values for the association tests were obtained from a χ^2 ^test unless the number of cases was fewer than five in any category, in which case, Fisher's Exact test was used.

One of the most important discoveries in breast cancer research in recent years has been the realization that estrogen receptor (ER)-positive and -negative breast cancers represent molecularly distinct diseases with large differences in gene-expression patterns [5,6]. Therefore, gene expression-based prediction of ER status represents an easy classification problem.

A somewhat more difficult problem is to predict extreme chemotherapy sensitivity, including all breast cancers in the analysis. This classification problem is facilitated by the association between clinical disease characteristics and chemotherapy sensitivity. For example, ER-negative cancers are more chemotherapy sensitive than are ER-positive tumors [7].

A third, and more difficult, classification problem is to predict disease outcome in clinically and molecularly homogeneous patient populations. Genomic predictors could have the greatest clinical impact here, because traditional clinical variables alone are only weakly discriminatory of outcome in these populations. In the current data set, prediction of chemotherapy sensitivity among the ER-negative cancers represents such a challenge.

The goal of this analysis was to assess how the degree of classification difficulty may affect which elements of prediction methods perform better. We divided the data into a training set (*n* = 130) and a validation set (*n* = 100) and developed a series of classifiers to predict (a) ER status, (b) pathologic complete response (pCR) to preoperative chemotherapy for all breast cancers, and (c) pCR for ER-negative breast cancers. A predictor, or classifier, in this article is defined as a set of informative features (generated by a particular feature-selection method) and a trained discrimination rule (produced by applying a particular classification algorithm).

First, we examined whether the success of a predictor was influenced by a feature-selection method. We examined five different univariate feature-selection methods including three variations of a *t *test-based ranking and two methods that order features based on differences in expression values. It has been shown that several different classification algorithms can yield predictors with rather similar performance metrics [8-10]. However, it remains unknown whether the relative performances of different methods may vary depending on the difficulty of the prediction problem. We examined this question for eight different classifiers representing a broad range of algorithms, including linear (LDA), diagonal linear (DLDA), and quadratic discriminant analysis (QDA); logistic regression (LREG); and two versions of support-vector machines (SVM) and k-nearest neighbor (KNN) methods. Altogether, 40 different predictors were developed for each of the three classification problems (five different feature-selection methods × eight different classifiers). We also were interested determine to what extent the cross-validation classification performance is influenced by different data-resampling methods and the difficulty of the classification problem. We estimated the classification performance by using 10-times-repeated fivefold cross validation (10 × 5-CV) and leave-pair-out (LPO) bootstrapping [11] (a method that better accounts for training and testing variability). We calculated performance metrics for each of the 120 predictors (40 predictors × three endpoints) and compared the estimated accuracy in the training set with the observed accuracy in the independent validation set.

## Materials and methods

### Patients and materials

Gene-expression data from 230 stage I to III breast cancers, without individual patient identifiers, were provided to the MAQC project by the University of Texas M.D. Anderson Cancer Center (MDACC) Breast Cancer Pharmacogenomic Program. Gene-expression results were generated from fine-needle aspiration specimens of newly diagnosed breast cancers before any therapy. The biopsy specimens were collected sequentially during a prospective pharmacogenomic marker discovery study approved by the institutional review board between 2000 and 2008. These specimens represent 70% to 90% pure neoplastic cells with minimal stromal contamination [12]. All patients signed informed consent for genomic analysis of their cancers. Patients received 6 months of preoperative (neoadjuvant) chemotherapy including paclitaxel, 5-fluorouracil, cyclophosphamide, and doxorubicin, followed by surgical resection of the cancer. Response to preoperative chemotherapy was categorized as a pathologic complete response (pCR = no residual invasive cancer in the breast or lymph nodes) or residual invasive cancer (RD). The prognostic value of pCR has been discussed extensively in the medical literature [13]. Genomic analyses of subsets of this sequentially accrued patient population were reported previously [9,14,15]. For each endpoint, we used the first 130 cases as a training set to develop prediction models, and the next 100 cases were set aside as independent validation set. Table 1 and Additional file 1 show patient and sample characteristics in the two data sets.

### Gene-expression profiling

Needle-aspiration specimens of the cancer were placed into RNAlater™ solution (Qiagen, Germantown, MD, USA) and stored at -80°C until further analysis. RNA extraction and gene-expression profiling were performed in multiple batches over time, as described previously [16,17] by using Affymetrix U133A (Affymetrix, Santa Clara, CA, USA) microarrays. Gene-expression data have been uploaded to the Gene Expression Omnibus website under the accession number GSE16716. Normalization was performed by using MAS 5.0 software (Affymetrix, Santa Clara, CA, USA) with default settings. Quality-control assessment of the hybridization results were performed with SimpleAffy software by Bioconductor; the percentage present call had to be more than 30%, scaling factor less than 3, and the 3'/5' ratios for β-actin less than 3, and for GAPDH, less than 1.3. These quality-control metrics are presented for each case in Additional file 2.

### Ranking of classification problems by informative feature utility score

To assess the relative difficulty of the three classification problems that we selected to study, we adopted an approach similar to that described in [18]. This method defines the utility of a feature *i *as its Fisher score,

where *μ*_1*i *_and *μ*_2*i *_are the class means, and *σ*_1*i *_and *σ*_2*i *_are the class standard deviations for the feature *i*, respectively. If features are ordered *f*_1 _≥ *f*_2 _≥ ... then, for each endpoint, the cumulative information is defined as

where N is the sample size. This cumulative information score assumes that the features are independent and that their effect on the classification performance is additive. This is rarely the case, as features are often correlated. Nonetheless, this cumulative information score is a simple and straightforward approach to estimate the relative difficulty of a classification problem early in the classifier-development process: an easier problem tends to have larger values for *F *than does a more difficult problem.

### Feature-selection methods

No prefiltering of probe sets was done; all probe sets were considered by the feature-ranking methods that included (a) unequal variance *t *test (FS1); (b) unequal variance *t *test with filtering of probe sets that were correlated with one another (Pearson correlation > 0.75) to generate independently informative features (FS2); (c) instead of removing the correlated features, they were combined into meta-features by averaging them (FS3); and (d) we also ranked features according to their ratio of between- to within-group sum of squares (FS4) and (e) according to the absolute differences in the class means (FS5).

### Classification algorithms

We examined eight classifiers in combination with the previously mentioned feature-selection methods, including linear discriminant analysis (LDA), diagonal linear discriminant analysis (DLDA), quadratic discriminant analysis (QDA), logistic regression (LREG), two k nearest neighbors classifiers with k = 3 (KNN3) and k = 11 (KNN11), and support vector machines with a radial basis function kernel with two different values for the kernel parameter: γ = 0.5 (SVM05) and γ = 2.0 (SVM2), respectively. Overall, 40 models were developed for each of the three prediction problems.

### Estimation of predictive performance

Leave-N-out cross-validation and other resampling methods of the training set are often used to select a final predictor for independent validation. Therefore, it is important to understand how resampling-based predictive performance correlates with predictive performance on independent validation cases. To study this question, we used a nested two-level cross-validation scheme, in which the cross-validation in the outer loop had the role of estimating the performance of the whole modeling procedure, whereas the cross-validation in the inner loop was used for selecting the optimal number of features [19].

The procedure in the inner loop is as follows. For each combination of a feature-selection method F and a classification algorithm C, the number of features j(F, C) in the model was considered as a free-parameter (within a predefined set of allowable values) and was optimized. In the inner loop, a repeated (5 times), stratified (to preserve the proportion of the two classes in all training and testing splits), fivefold cross-validation was used to define the number of features that maximized the AUC. A ranking of the features was first obtained by applying *F *on the reduced internal training set (obtained by leaving aside one fold from the current training set). Then the classifier *C *was trained on the same set, but considering only the top j(*F, C*) features. The predictions on the internal testing set (the left-out fold) were recorded, and the procedure was repeated. At the end, an estimation of the AUC was obtained, corresponding to the given combination of *F, C*, and j(*F, C*). The procedure was repeated with different folds, and an average estimate of the AUC was obtained for each *F, C*, and j(*F, C*). The optimal number of features j*(*F, C*) was selected as the value j(*F, C*) yielding the highest average AUC. The number of features allowed for each model was chosen *a priori*, to avoid overfitting of models and to limit the computation time. For the prediction of ER status, the feature size was chosen to contain all values between 2 and 15, whereas for both pCR endpoints, it was {2,5,8,...,41}; 41 being almost half the size of the smallest training set (*n* = 85 ER-negative cancer). For a pseudo-code that details the schema used for cross-validation [see Additional file 3]. To avoid adding variability due to random partitioning the data into folds, all estimates were obtained on the same splits of the data.

We investigated two methods in the outer loop. The first method is a stratified 10-times-repeated fivefold cross-validation (10 × 5-CV). In each of the five cross-validation iterations, 80% of the data were first used as input to the inner loop procedure for feature selection and training the classifier with the selected features, and finally, the remaining 20% of the data were used to test the classifier. The 95% CI for the area under the receiver operating characteristics curve (AUC) was approximated by [AUC - 1.96 SEM, AUC + 1.96 SEM]. The SEM was estimated by averaging the 10 estimates of the standard error of the mean obtained from the five different estimates of the AUC produced by the 5-CV.

The second method in the outer loop is a bootstrap-based method, also known as a smoothed version of cross-validation [20]. Efron and Tibshirani [20] proposed the leave-one-out bootstrap method on the performance metric error rate, and their technique was recently extended by Yousef and colleagues [11] to the performance metric AUC. This method uses a leave-pair-out (LPO) bootstrap approach to estimate the mean AUC (mean over training sets) and a "delta method after bootstrap" to estimate the variability of the estimated mean AUC. We point out that this variability captures both the effect of finite training-set size and the effect of finite testing-set size. In the LPO approach, multiple (*n* = 5,000) training sets are obtained by stratified bootstrap resampling, and each training set is used as input to the inner-loop procedure for feature selection and training the classifier with the selected features. In testing, any pair of cases (one from the positive class and one from the negative class) is tested on the classifiers trained on the bootstrap samples that do not contain the two held-out cases. The Wilcoxon-Mann-Whitney statistic of the prediction results on pairs of cases is averaged over all bootstrap-training sets and is used to estimate the mean AUC. An advantage of this technique is that it allows estimating the variability of the AUC estimator by using the influence function method [11,20]. By assuming that the estimated AUC is asymptotically normal, the 95% CI of the AUC can be approximated by [AUC - 1.96 SEM; AUC + 1.96 SEM].

The estimated performance and the associated CIs from the training and internal-assessment process are compared with the independent validation performance. The conditional validation performance was obtained by selecting features and training the classifier with the training data set and testing on the validation data set. This performance is conditional on the particular finite training set and may vary when the training set varies. Therefore, we estimated the mean of this conditional performance where the mean is over multiple training sets and obtained by bootstrapping the training set multiple times and averaging the conditional AUCs, as tested on the validation set [21].

We also estimated the variability of the conditional validation performance and decomposed the variance into two components: the variability due to the finite size of the training set and the variability due to the finite size of the test set [21]. The training variability reflects the stability of the classifier performance when the training set varies, and the testing variability reflects the expected performance variation for different test sets.

To compare the ability of the performance estimates of 10 × 5-CV and the LPO bootstrap to predict the performance on the independent set, we used a root mean square error (RMSE) measure, which is defined as

where *F *and *C *index feature selection and classifier, respectively,  denotes the mean AUC; the superscript "internal" can be "10 × 5-CV" or "LPO bootstrap."

### Estimation of predictor learning over increasing training-set size

Predictor learning was evaluated for the models that performed nominally the best in independent validation for each of the three prediction problems. All 230 cases were included in the analysis to fit learning curves to these three models. For the ER-status endpoint, 10 different training-sample sizes, ranging from *n* = 60 to *n* = 220 by increments of 20, were used to estimate the dependence of the performance parameters on the sample size. For each sample size, 10 different random samples were drawn from the full set by stratified sampling, and fivefold cross-validation was used to assess the error rate and AUC of the models where all the parameters of the models were recalculated. A similar approach was taken for the pCR (*n* = 50, 70, ..., 210) and "pCR in ER-negative cancer" predictors (*n* = 25, 40, ..., 85). By following the work of Fukunaga [22], the following learning-curve model was fit to the resulting AUC: *Y *= *a*+*b*/*TrainingSize*.

### Congruence of different predictors at gene and functional pathway level

We were interested in examining the congruence of informative features that were selected by different methods for the same prediction endpoint and also for different endpoints. Both gene-level and pathway-level analyses were performed as described previously [23]. MetaCore protein-function classification was used to group genes into protein functions, and GeneGo Pathway maps were used for functional classification of predictive features. We assessed congruency by using the kappa statistics. The input for kappa involves "learners" that classify a set of objects into categories. We considered each feature-selection method as a learner and each probe set as an object. The probe sets used in this analysis are presented in Additional file 4. Each probe set from the rank-ordered lists is categorized by each feature-selection method either as 1 (that is, selected as informative) or 0 (that is, nonselected). By using such an 0/1 matrix for all probe sets × all feature-selection methods for every prediction endpoint as input, we can calculate Cohen's kappa function for the congruency. For pathway-level analysis, we mapped the probe sets to pathway lists by using hypergeometric enrichment analysis. The pathways are ranked by enrichment *P *values, and the top n pathways (n equals the number of genes in the input list for comparison and consistency between the two levels) were selected for presentation.

All statistical analysis was performed by using R software.

## Results

### Difficulty of the classification problems

**Figure 1:**
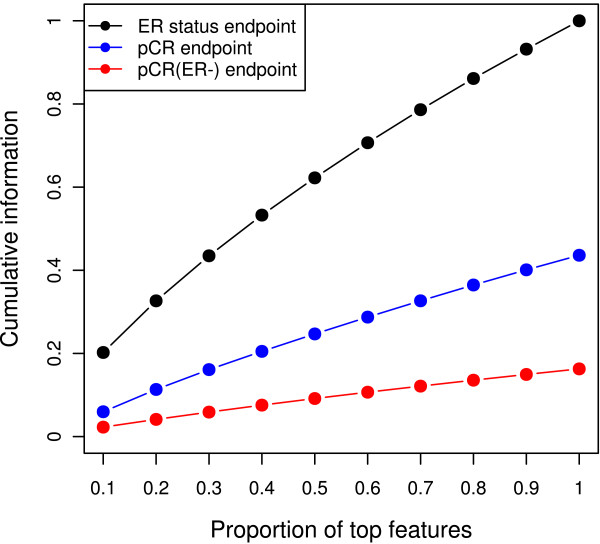
**Relative complexity of the three prediction problems**. The cumulative information values have been scaled such that the maximum value is 1. To make the curves comparable and to take into account the sample size, the ratio between the number of features used in the cumulative information (F) and the sample size is used on the horizontal axis. Larger values of the cumulative information indicate simpler problems.

Three distinct classification problems were studied: (a) ER-status prediction, including 80 ER-positive (62%) and 50 ER-negative training cases (38%); (b) pCR prediction, including 33 cases with pCR (25%) and 97 cases with residual cancer (75%) for training; and (c) pCR prediction for ER-negative cancers, including 27 training cases with pCR (54%) and 23 with residual cancer (46%). Figure 1 shows the cumulative information scores for the three endpoints: larger cumulative information is an indicator for a simpler classification problem. The obtained ranking implies that the three endpoints represent different degrees of classification difficulty.

We also assessed the significance of the utility scores by using permutation tests (10,000 permutations) for computing the raw *P *values, followed by Benjamini-Hochberg correction for multiple testing. For the ER-status endpoint, 1,502 features with significant utility scores (*P *value < 0.0001) were used, whereas for the pCR (all cases), 252 significant features and only five features (corresponding to A2M [HGNC:7], RNMT [HGNC:10075], KIAA0460 [HGNC:29039], AHNAK [HGNC:347], and ACSM1 [HGNC:18049] genes) were used for pCR among ER-negative cancers.

### Effect of feature-selection methods and classification algorithms on cross-validation performance

**Figure 2:**
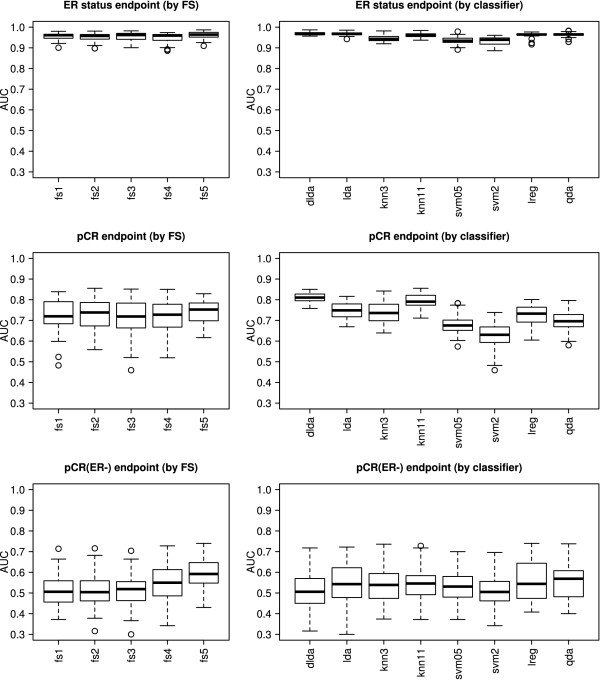
**Boxplots of the estimated area under the curve (AUC), stratified by feature-selection and classification methods**. The boxplots show the mean AUC in 10 times fivefold cross validation (CV). The left column contains the estimated AUC stratified by the feature-selection method, and the right column contains the estimated AUC stratified by the classification method.

Figure 2 illustrates the average cross-validation AUC estimated by 10 × 5-CV for all predictors, stratified by feature-selection method (left column). All feature-selection methods performed similarly in combination with various classification algorithms for a given endpoint. The two non-*t *test-based methods, FS4 and FS5, showed slightly better performances than did *t *test-based feature selection for the most difficult prediction endpoint "pCR on ER-negative cancers" in cross validation, but confidence intervals widely overlapped. Additional file 5 shows the average error rates and AUCs generated from 10 × 5-CV for each prediction model applied to all three classification problems, along with the average number of features selected. Interestingly, the number of selected features did not increase as the prediction problem became more difficult. For the most difficult problem, the number of selected features was lower than that for the moderately difficult problem. This is probably because of the lack of informative features: as the classification problem becomes more difficult, fewer features are informative for the outcome (also see Figure 1).

Figure 2 also shows the variability of the classification error rates and AUC estimated through 10 × 5-CV for all predictors, stratified by classification algorithm (right column). All methods performed similarly. The prediction endpoint (that is, classification difficulty) had the greatest effect on the cross-validation AUC. The effects of feature-selection method and choice of classifier algorithm were modest.

### Bootstrap and independent-validation results

**Figure 3:**
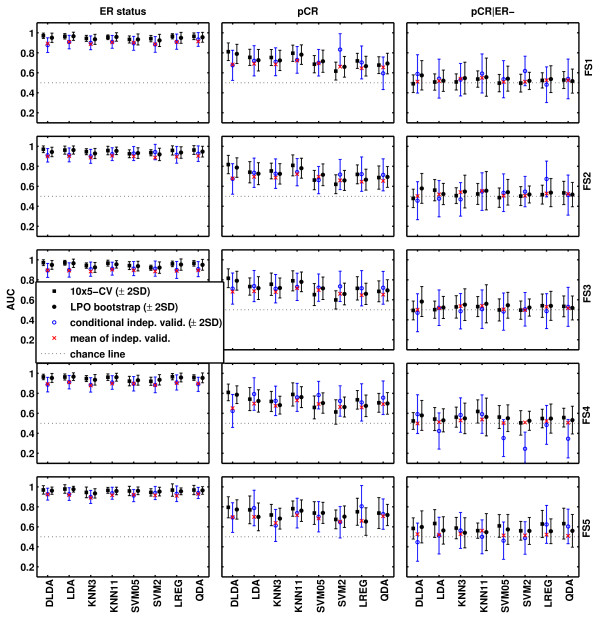
**Graphic summaries of the estimated and observed areas under the curve (AUCs) for each of the 120 models**. For each combination of feature-selection method and classification algorithm, the AUCs ± 2 standard deviations are plotted. Mean AUCs obtained from 10 × 5-CV (cross-validation; black square), LPO bootstrap (black dot), and the conditional (blue circle) and mean (red cross) validation AUCs are shown.

Figure 3 shows the estimated AUCs obtained with 10 × 5-CV (black square), LPO bootstrap (black circle), and the conditional AUC (blue circle) on the independent validation set and its variability (blue error bar representing ± 2 SD) and mean (red cross). Additional file 5 includes the internal (10 × 5-CV and LPO bootstrap) and independent validation-performance metrics for each predictor. Both internal-estimation methods yielded AUCs that were very close, well within 2 standard deviations of the mean, to the conditional and mean AUCs observed in the independent validation. Internal-performance estimates generated within the training set only slightly overestimated the performance relative to independent validation, indicating both that the modeling approach was correct and that no strong batch effect occurred between training and validation sets. Simpler linear methods, such as LREG, LDA, and DLDA, performed generally well in both internal and independent validation, and these methods were among the top five nominally best-performing models for all prediction endpoints [see Additional file 5]. The non-*t *test-based feature-selection methods (FS4, FS5) that showed good results in cross validation also performed well in independent validation and were included in four of the top five models for each endpoint. However, the 95% CIs of the point estimates overlap broadly for all predictors, and no single strategy emerged as clearly superior for any particular endpoint.

To assess the confidence-interval estimation, we calculated the RMSE for the AUC estimates obtained with 10 × 5-CV and LPO bootstrap for all the three endpoints. Leave-pair-out bootstrap performed better than 10 × 5-CV in terms of the agreement with the mean AUC estimated in the independent-validation set: RMSEs for LPO bootstrap were 0.0484, 0.0491, and 0.357 in comparison with 0.0562, 0.0713, and 0.449 for 10 × 5-CV for the ER status, pCR, and pCR within ER-negative endpoints, respectively.

Figure 3 clearly shows that the variability of the estimated classification performance increases as the level of classification difficulty increases. This implies that, to achieve the same level of statistical precision of the estimated performance, more cases are needed for a more-difficult endpoint. Figure 3 also shows both the conditional (blue circle) and mean validation AUCs (red cross). The larger the difference between the conditional validation AUC and the mean validation AUC, the less stable the predictor is with respect to varying the training sets. A quantitative measure of classifier stability is the training variability, and we have decomposed the variability of the conditional validation AUC shown in Figure 3 into two components (training variability and testing variability) and put the results in Additional file 5.

### Predictor-performance and sample-size estimations through learning curves

**Figure 4:**
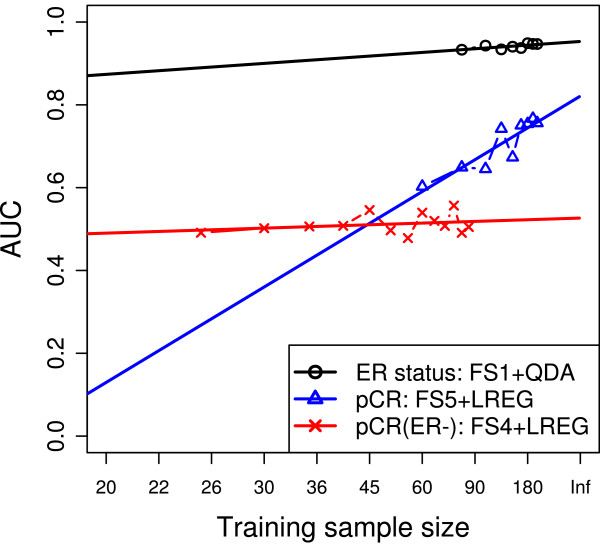
**Learning curves for the best predictors for each of the three endpoints**. For each endpoint, the learning curve of the best-performing model on the validation set was estimated by fivefold cross-validation for gradually increasing sample sizes. The plot shows both the estimated performance for different sample sizes and the fitted curve. The quadratic discriminant analysis (QDA) classifier required more than 60 samples, so the minimum sample size for it was 80. Note the nonlinear scale of the x-axis.

To estimate the training-set size that is necessary to develop predictors that operate near their respective plateaus, we examined how the performance characteristics of each of the nominally best predictors for each endpoint improved as the training-set size increased. For ER-status prediction, we selected QDA with FS1 (conditional validation AUC = 0.939); for pCR prediction including both the ER-positive and -negative cancers, we selected LREG with FS5 (conditional validation AUC = 0.805); and for pCR in ER-negative cancers, we selected LREG with FS4 (conditional validation AUC = 0.627). Figure 4 shows the observed changes in average AUCs for each of the classifiers as the training-set size increased from 60 to 220 (or from 25 to 85 for pCR prediction in ER-negative cancers) and the projected improvements for assumed larger training sets. The results indicate that for the easiest problem (ER), the predictor seems to perform at its best with a sample size around 80 to 100. For the moderately difficult problem (pCR), the steady increase of the learning curve suggests that the performance of the model can be improved by increasing the sample size, beyond the highest value currently tested (220). For the pCR in ER-negative cancer endpoint, the learning curves manifested a very modest and gradual improvement in performance between training sample sizes of 25 and 85, suggesting that either too few samples were available for a reliable estimation of the learning curve or that limited information in the mRNA space exists to predict this particular outcome with the methods applied in this analysis. The learning curve that had a slope significantly different from 0 was the one for the pCR endpoint (*P *= 0.001; ER endpoint, *P *= 0.05; pCR in ER-negative endpoint, *P *= 0.365).

### Functional analysis of predictive features

Our results demonstrate that several different feature sets can yield predictors with statistically similar performances [8-10,24]. This may occur because the various probe sets that represent different genes capture information from the same complex molecular pathways that determine a particular clinical outcome [25]. In other words, different features measure different components of the same informative biologic pathway. To test this concept, we mapped each of the 15 feature sets used in the final validation models to known biologic pathways. The different feature sets selected for a particular prediction endpoint had a high level of congruency at both the gene and the pathway levels across all the five different ranking methods (Table 2). The selected gene sets and pathways were also rather similar to each other for the ER and pCR prediction endpoints. However, the genes and pathways predictive of pCR in ER-negative cancers were very different from the other two informative gene sets.

**Table 2:** Congruencies across different endpoints and different feature-selection methods

Same endpoint but different feature selection (FS)		
Endpoint	Gene-level	Level of canonic-pathway maps
ER status	0.541	0.573
pCR	0.544	0.572
pCR(ER^-^)	0.593	0.532

**Same FS but different endpoints**		
**FS**	**Gene-level**	**Level of canonic-pathway maps**

FS1	0.300	0.290
FS2	0.299	0.274
FS3	0.291	0.278
FS4	0.295	0.291
FS5	0.272	0.282

The table shows that kappa statistics (that is, congruency) are high for different feature-selection methods for the same endpoint but are low for the same feature-ranking method for different endpoints. Both gene-level and pathway-level analyses show similar results.

Additional file 6 contains the pathway-enrichment tables for the three endpoints, including pathways with enrichment *P *values < 0.1. Thirty-two pathways contributed to the prediction of ER status; 36, to pCR prediction; and 11, to pCR prediction within ER-negative cancers across the five feature-selection methods. For the ER endpoint, development, cell adhesion, cytoskeleton remodeling, DNA damage, apoptosis, and ER transcription factor activity were the most significant pathway elements common to all informative feature sets. We also noted that most pathways that were involved in pCR prediction (31 of 36) were the same as those involved in ER-status prediction. This is consistent with the known association between pCR rate and ER status [7]. Estrogen receptor-negative cancers had significantly higher pCR rates than ER-positive cancers (54% pCR in ER-negative cancers versus 7.5% pCR in ER-positive cancers; χ^2 ^test *P *value = 1.068e-08). The pathways that were selected for prediction of pCR in ER-negative cancers were distinct from the pathways that were predictive of pCR in all patients and included immune response-related pathways (IL-2 and T-helper cell activation), opioid-receptor signaling, and endothelial cell-related pathways.

## Discussion

The goal of this analysis was to examine how the choice of a univariate feature-selection method and classification algorithm may influence the performance of predictors under varying degrees of classification difficulty. We examined the influence of changing two critical components, feature selection and classification algorithm in the predictor development process, for three different prediction problems that represented three levels of difficulty in a clinically annotated human breast cancer data set. Classification of breast cancer into ER-positive or -negative categories is an easy classification problem; the large number of informative probe sets and high information content of the features allow clear separation of the groups. The AUC values for the 40 different prediction models for this endpoint ranged from 0.875 to 0.939 in the independent validation set. Prediction of pCR across all breast cancers, including both ER-negative and ER-positive cases, represented a slightly more difficult prediction problem with AUCs ranging between 0.61 and 0.80 in the validation set. Prediction of pCR in the molecularly more homogeneous ER-negative breast cancer subpopulaton proved to be the most difficult classification challenge: the validation AUCs ranged from 0.34 to 0.62. No predictor-development strategy emerged as clearly superior for any of the classification problems. The 95% CI of the prediction accuracies overlaped broadly for most of the predictors. However, LDA, DLDA, LREG, and QDA classification algorithms were consistently among the best-performing models for each problem. Interestingly, KNN3 and SVM methods were often among the worst-performing models in independent validation, even though these reached relatively high AUC values in cross validation. It is possible that further fine tuning of parameters for these more-complex classifiers (in the sense of an implementable decision boundary) could have improved predictive performance. We examined only the radial function kernel for SVM with two *a priori *set kernel parameters γ = 0.5 and 2.0, and the parameter C (cost of misclassification) was also fixed at 10. Fixing these parameters may have resulted in "less than optimally trained" models that could lead to added variability in the performance of the classifiers. Also, we examined only two versions of KNN with *a priori *set *k *of 3 and 11, and found that KNN11 outperformed KNN3. Low values of *k *yield local classifiers with low bias but high variance, whereas higher values led to more-global classifiers with higher bias and lower variance; exploring a broader range of *k *values could have optimized prediction results. Optimizing the parameters γ or *k *is not a straightforward task. It should be done within the inner cross-validation process, just as is done with feature selection. Fine tuning different model parameters outside of the two-stage cross-validation process would lead to model-selection bias, or optimization bias [19].

An interesting observation was that simple feature-selection methods that ranked features based on difference in means performed very well in both cross-validation and independent validation relative to the more commonly used *t *statistic-based ranking. Four of the top five models for each prediction problem used features selected by the non-*t *test-based methods. However, it is important to recognize that all of the feature-selection methods that we examined represented univariate filtering approaches that rank features individually and independent of the classification method. It is possible that nonparametric or multivariate feature-selection methods could yield different results. Penalized feature-selection methods, which embed feature selection in the classifier fitting step, may also have advantages, because features that might not be discriminatory individually could be jointly predictive in combination with other features. At least one article suggested that multivariate sparse penalized likelihood methods, including lasso and elastic net, might have a slight edge compared with univariate filtering [26]. Other publications that compared several univariate and multivariate feature-selection methods in public cancer data sets by using 10-fold cross-validation estimates found that simple univariate feature-selection methods often outperformed more-complex multivariate approaches [27,28].

Our data demonstrate that many different feature sets and classification methods can yield similarly accurate predictors for a given endpoint. When we mapped the feature sets generated by five different univariate feature-selection methods to biologic pathways, each method tended to identify similar genes and pathways. The biologic pathways that were implicated in ER-status or pCR prediction were distinct from the pathways that were predictive of pCR in ER-negative cancers. This pathway-level analysis is hypothesis generating and will require further laboratory validation to determine the importance of the identified pathways (for example, immune response, endothelial-cell regulation, G-protein signaling) in the biology of chemotherapy response in ER-negative breast cancer.

To estimate potential improvements in predictive performance of the nominally best predictors for each classification problem, we pooled all cases and carried out a series of split-sample training and validation analyses in which the predictors were trained on increasingly larger data sets. For the easy classification problem (ER-status), relatively small sample sizes (80 to 100 samples) were enough for constructing excellent predictors. In contrast, for the moderately difficult problem (pCR prediction), the accuracy of the model steadily improved as the sample size increased. For the most difficult problem, pCR prediction in ER-negative cancer, a minimal improvement was observed over a range of 25 to 85 training cases. It is important to note that the pCR and ER status predictors trained on 80 cases showed good or excellent conditional AUCs (0.65 and 0.94, respectively). This modest performance and limited improvement of the pCR predictor for ER-negative cancer may be due to (a) too small sample size for trainig or (b) the incompletness of the mRNA expression-based feature space, meaning that this class-separation problem cannot be fully accomplished by using information only from the available probes by using the methods that we applied. However, fitting learning curves to preliminary data sets could assisst investigators in estimating sample-size requirements for a particular prediction problem for any given model.

## Conclusions

This analysis confirms that it is possible to build multigene classifiers of clinical outcome that hold up in independent validation. Predictor performance is determined largely by an interplay between training-sample size and classification difficulty. Variations on univariate feature-selection methods and choice of classification algorithm had only a modest impact on predictor performance, and it is clear that within our statistical precision, several equally good predictors can be developed for each of our classification problems. Pathway-level analysis of informative features selected by different methods revealed a high level of congruency. This indicates that similar biologic pathways were identified as informative for a given prediction endpoint by the different univariate feature-selection methods. The independent validation results also showed that internal 10 × 5-CV and LPO bootstrap both yielded reasonably good and only slightly optimistic performance estimates for all the endpoints.

## Abbreviations

10 × 5-CV: repeated (10 times) fivefold cross validation; AUC: area under the receiver operating characteristic curve; CI: confidence interval; DLDA: diagonal linear discriminant analysis; ER: estrogen receptor; KNN: k nearest-neighbors classifier; LDA: linear discriminant analysis; LPO: leave-pair-out bootstrap; LREG: logistic regression classifier; pCR: pathologic complete response; QDA: quadratic discriminant analysis; RD: residual invasive cancer; RMSE: root mean square error; SD: standard deviation; SEM: standard error of the mean; SVM: support vector machine.

## Competing interests

The authors declare that they have no competing interests.

## Authors' contributions

LP, VP, and LS designed the study. VP, WFS, and WC performed the experiments. VP, WC, BG, CH, WS, FS, YN, MT, AI, TN, KH, MD, and LP performed the statistical analyses and interpreted the results. VV, DB, GH, WFS, and LP contributed the clinical, pathologic, and molecular data. All authors contributed to the writing of the manuscript and read and approved the manuscript.

## Supplementary Material

Additional file 1 **Supplemental Table S1**. Clinical data for all the patients in the training and validation sets. Click here for file

Additional file 2 **Supplemental Table S2**. Quality control results. Click here for file

Additional file 3 **Supplemental Table S3**. Pathways mapping for all endpoints. Click here for file

Additional file 4 **Supplemental methods**. Pseudo-code description of the two-level external cross-validation scheme. Click here for file

Additional file 5 **Supplemental Table S4**. Features (probesets) selected in the 120 models. Click here for file

Additional file 6 **Supplemental Table S5**. Estimated and validation performance of all models. Click here for file
