# Supplementary material for: Effect of training-sample size and classification difficulty on the accuracy of genomic predictors
Source: Breast Cancer Res. 2010 Jan 11;12(1):R5. doi: 10.1186/bcr2468 (PMC2880423; doi:10.1186/bcr2468)
Supplement: Additional file 4 — Supplemental methods. Pseudo-code description of the two-level external cross-validation scheme. [file bcr2468-S4.pdf]

Supplemental methods 1: Pseudo-code for the two-level external cross-validation scheme.

```
Input:
  -expression matrix X and the
corresponding label vector y;
  -feature selection method F;
  -classifier C;
  -set of candidate numbers of features;
  -the number of repeats R;
  -the number of folds K;
Output: performance estimation


For r = 1,…,R do:
  For k = 1,…,K do:
    -generate the current training set by
discarding the k-th fold:
    X_tr = X \ X^(k); y_tr = y \ y^(k);
    -find the optimal number of features:

    nf = optimal_number_of_features (X_tr,
y_tr, F, C, S);

    -select top nf features according to
the feature selection method F:
    Z = get_top_features(X_tr, y_tr, F,
nf);
    -build the current model:
    model = train_classifier(Z, y_tr, C);
    -predict the labels for y^(k) and save
them;
  End for
  -use the save predictions for measuring
the performance (error rate, AUC, …)
End for

Return performance statistics (average
error rate, average AUC, …)
```

```
Function
optimal_number_of_features(X,
y, F, C, S):

For r = 1,…,R do:
  For k = 1,…,K do:
    -generate the current
training set by discarding the
k-th fold:
    X_tr = X \ X^(k); y_tr = y \
y^(k);
    For n in S do:
      -select top nf features
according to the feature
selection method F:
      Z =
get_top_features(X_tr, y_tr, F,
n);
      - build the current
model:
      model =
train_classifier(Z, y_tr, C);
      -predict the labels for
y^(k) and save them;
    End for
  End for
  -from the predicted labels
compute the area under the ROC
curve for every n and for
```

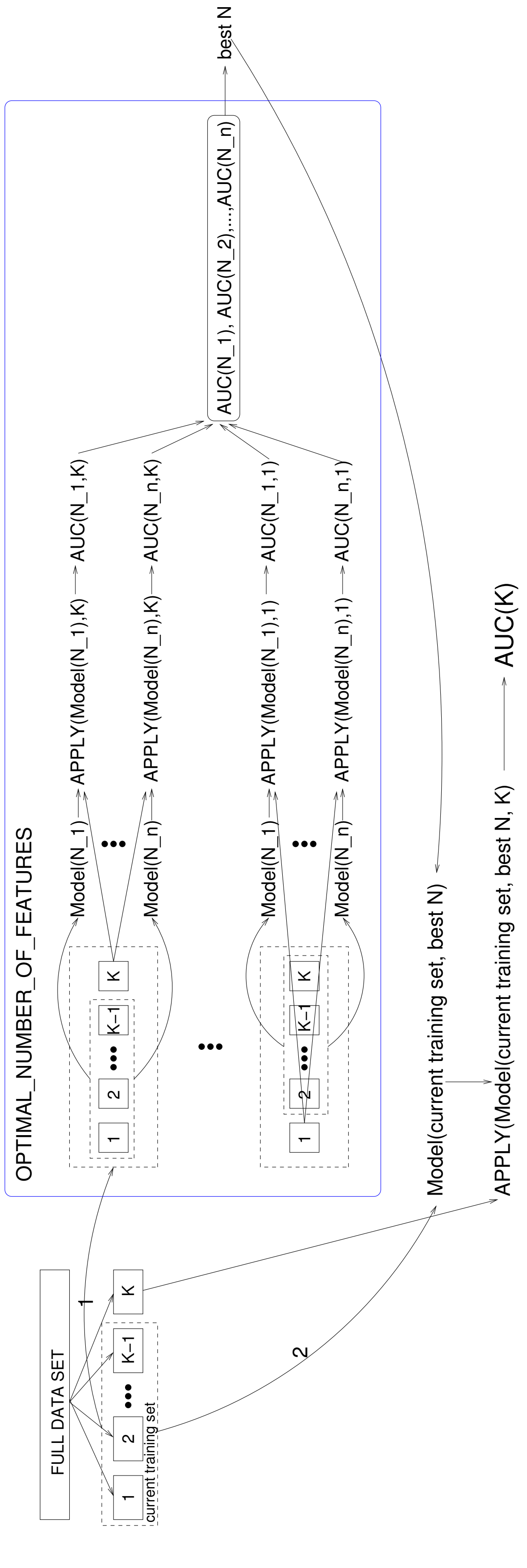

Schematic representation of the nested cross-validation approach. For a given partition of the full data set in the outer cross-validation, an inner cross-validation is performed to estimate to optimal number of features (the one that maximizes the AUC). Then, in the outer cross-validation, a model with the optimal number of features is constructed on the current training set and it is apply on the left-out fold (here the K-th). Repeating this scheme leads to K estimates of the performance, which are averaged to obtain the final performance estimation.
